# Improving usability of Electronic Health Records in a UK Mental Health setting: a feasibility study

**DOI:** 10.1007/s10916-022-01832-0

**Published:** 2022-06-08

**Authors:** Ruta Buivydaite, Gurpreet Reen, Tatjana Kovalevica, Harry Dodd, Ian Hicks, Charles Vincent, Daniel Maughan

**Affiliations:** 1 Department of Experimental Psychology, University of Oxford, Oxford, UK; 2 Oxford Health NHS Foundation Trust, Oxford, UK

**Keywords:** Electronic Health Records, Usability testing, Usability improvements, Mental Health

## Abstract

**Background:**

Electronic Health Records (EHRs) can help clinicians to plan, document and deliver care for patients in healthcare services. When used consistently, EHRs can advance patient safety and quality, and reduce clinician’s workload. However, usability problems can make it difficult for clinicians to use EHRs effectively, which can negatively impact both healthcare professionals and patients.

**Objective:**

To improve usability of EHRs within a mental health service in the UK.

**Methods:**

This was a feasibility study conducted with two mental health teams. A mixed-methods approach was employed. Focus group discussions with clinicians identified existing usability problems in EHRs and changes were made to address these problems. Updated EHR assessment forms were evaluated by comparing the following measures pre and post changes: (1) usability testing to monitor time spent completing and duplicating patient information in EHRs, (2) clinician’s experience of using EHRs, and (3) proportion of completed EHR assessment forms.

**Results:**

Usability testing with clinicians (n = 3) showed that the time taken to complete EHR assessment forms and time spent duplicating patient information decreased. Clinician’s experience of completing EHR assessment forms also significantly improved post changes compared to baseline (n = 71; p < 0.005). There was a significant increase in completion of most EHR forms by both teams after EHR usability improvements (all at p < 0.01).

**Conclusions:**

Usability improvements to EHRs can reduce the time taken to complete forms, advance clinician’s experience and increase usage of EHRs. It is important to engage healthcare professionals in the usability improvement process of EHRs in mental health services.

**Supplementary information:**

The online version contains supplementary material available at 10.1007/s10916-022-01832-0.

## Introduction

Electronic Health Records (EHRs) are a “repository of patient data in digital form, stored and exchanged securely” and an important feature of modern healthcare systems [1]. EHRs can help healthcare professionals to plan, document and deliver care for their patients, as well as exchange information with other healthcare providers to provide continuity of care. Consistent use of EHRs can also reduce the rate of medical errors, improve patient safety and quality, as well as improve organisational efficiency [2–5]. However, if clinicians find EHRs disorganised or complex to use, then they may not be completed consistently which can diminish the benefits associated with EHRs and actively contribute to patient harm [6].

### Usability problems in EHRs

Usability or ease of use predicts whether a technological system such as EHRs will be accepted and used consistently [7]. This is supported by the technology acceptance model which states that the ease of use, defined as whether the user can use the system without too much effort, alongside the system’s perceived usefulness can predict the behavioural intention of whether the system will be used and adopted [8, 9]. Studies also show that clinicians report usability problems to be the most common barrier in using EHR systems [10, 11]. Difficulties in completing EHRs due to usability problems can further have implications for both clinician’s and patient’s well-being. Usability problems in EHRs can increase clinician’s cognitive workload [12], as well as increase time pressures and psychological distress [13]. The extra burden on clinical staff can also limit the time clinicians have available for direct patient care [14]. Improper use of EHRs due to usability problems can have a negative impact on the accuracy and quality of patient record keeping, which can subsequently lead to poor quality patient care [4, 15].

### EHRs usability in mental health

There has been little research conducted on EHRs usability in mental health [16, 17]. EHRs are used to record sensitive and potentially stigmatising patient information within the mental health context [16, 18]. However, EHRs usability problems can prevent proper completing of patient records and distress mental health patients who may be expected to repeatedly re-live traumatic experiences as their information is not properly recorded [16, 19]. Improving EHRs usability in mental health contexts is particularly challenging as healthcare professionals may have different and at times conflicting requirements from EHRs depending on their clinical teams [16, 17]. Some of these difficulties could be overcome by tailoring the EHR system based on common needs of mental health teams, ideally by involving clinicians throughout this process [17, 20, 21].

### Improving EHRs usability

Studies that improve EHRs usability found better clinician satisfaction with the system and improvements in clinician’s cognitive workload and performance [12, 22]. Examples of usability improvements included customising EHRs for different clinical professions, as well as adding navigational pathways, keyboard shortcuts and auto-population patient records which can pull information from other sections of the EHR system [12, 17, 22]. Feasibility testing and clinician participation at all stages of EHR improvement have further been advocated as an approach to improve usability of the system for healthcare professionals [13, 17, 23].

### Methods to evaluate usability improvements

Clinician-based surveys are commonly used to evaluate usability improvements [7, 24–27]. However, many studies do not use validated surveys or describe survey development. Objective measures, such as capturing completed patient information in EHRs, has also been recommended [1, 4]. Usability testing is another objective measure which can help indicate whether a system is efficient, effective and easy to use for the intended user [28, 29]. This testing allows users to perform realistic tasks using the system in typical conditions, during which the task completion rate, mouse clicks or time taken to complete tasks is recorded [15, 24, 29, 30]. A combination of objective and self-report measures to evaluate usability improvements is likely to be the most informative.

### Aim

The aim of the current study was to improve the usability of EHR assessment forms completed after a mental health assessment within a clinical mental health setting in the UK. This was a feasibility study conducted with two mental health teams prior to implementation across the wider organisation. It was hypothesised that improving usability of EHR assessment forms in collaboration with clinicians will: (i) increase the number of completed forms, (ii) improve overall clinician experience, and (iii) reduce the time spent completing and duplicating patient information.

## Methods

### Study design

The present study evaluated usability changes made to existing EHR assessment forms in a pre and post design using three forms of measurement.

### Setting and participants

Two community-based adult mental health teams were conveniently sampled based on availability for feasibility testing from one mental health NHS Trust in the UK. Both teams provide community mental health care for about 1000 adult patients and employs around 50 multidisciplinary staff including psychiatrists, social workers, occupational therapists and community psychiatric nurses.

### Procedure

Usability changes were made to existing EHR assessment forms which are typically completed following a mental health assessment with a patient (see Box 1). These EHR assessment forms have been in use within the healthcare organisation since 2015, replacing a previous version of EHRs.

**Fig. 1:**
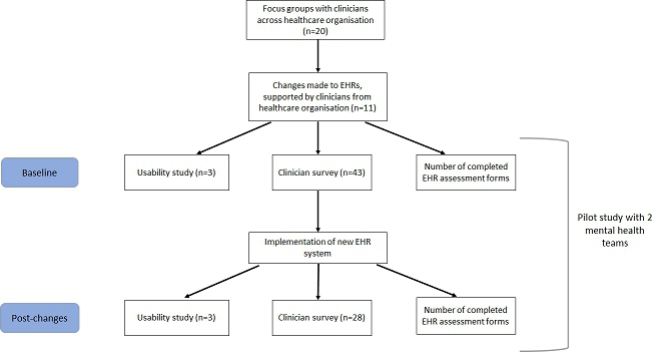
Flow chart summarising study procedure

Usability improvements to EHR assessment forms were made by the EHR team, in collaboration with clinicians. First, a focus group was conducted with clinicians to identify common usability barriers, followed by iterations to the EHR assessments with regular consultation from clinicians across the two teams. This process took approximately six months. The new EHR assessment forms were trialled on the two mental health teams on 15th April 2019. Baseline testing was conducted in the two months before implementation, and post-testing was conducted within ten weeks after implementation (i.e. the pre-post evaluation phase was between 18th February 2019 and 23rd June 2019). See the flow-diagram in Fig. 1 for a summary of the study procedure.

Box 1. EHR assessment forms to complete following a routine mental health assessment in a UK mental health service

- Mental health assessment: A record of the clinical assessment of a person's mental health condition
- Physical health assessment: A record of physical health conditions, medication, smoking, alcohol and drug use and cardiovascular risk factors.
- Risk assessment: A record of risks associated with the clinical case
- Health of the Nation Outcome Scale (HONOS): A scale containing 12 items measuring behaviour, impairment, symptoms and social functioning
- Patient care plan: A record of the plan of care for the person
- Letter to the primary care clinician: A letter for the patient’s primary care clinician recording key information from the mental health assessment

**Development of updated EHR assessment forms**

**Focus group**

A focus group was conducted with 20 clinicians from across the healthcare organisation to identify common usability barriers of using EHR assessment forms. Three members of the EHR team were also present. The most common usability barriers reported were: time taken to complete forms, duplicating when entering patient information and difficulty navigating the system (see Table 1).

**Table 1:** Common usability barriers to using EHR assessment forms as reported by clinicians in focus group

Usability barrier	Examples from focus groups
Unrelated questions on assessment forms	Clinicians from child and adolescent mental health teams found that some questions on the assessment forms were not relevant to the younger population. The teams also noted that several important questions for the younger population were not included on the assessment forms in the EHR system (e.g. safeguarding). Similar issues were reported by clinicians from other specialist teams.
Time taken to complete forms	Completing assessment forms on the EHR system was found to be very time-consuming due to the difficulty in navigating the system, as well as duplication from paper-based forms and within the system.
Duplicate questions within assessment forms	Clinicians highlighted that many questions were duplicated across the risk assessment and physical health assessment forms on the EHR system. This increased the time taken for clinicians to complete the forms.
Difficulty navigating the EHR system	Many clinicians found it difficult to navigate the EHR system to access relevant assessment forms. This further increased the time taken for clinicians to complete the forms.

**Iterations of EHR assessment forms**

The EHR team made usability changes to the EHR assessment forms based on the common usability barriers identified in the focus group. These changes were further refined with regular input from 11 clinicians representing the two mental health teams. Clinicians provided feedback to the EHRs team about the questions that should be present on the assessment form, on question order and question format, and how EHR assessment forms could best be used to auto-populate a letter to the primary care clinician.

**Updated EHR assessment forms**

The final changes made to the EHR assessment forms were: adding conditional logic, removing duplicate questions, auto-population of future assessment forms, auto-population of letters to primary care clinicians and a visual workflow dashboard (see Table 2 and appendix Figure A.1-A.3).

**Table 2:** Finalised changes to the EHR assessment forms

Usability barrier	Change made to EHR system	Assessment forms affected	Summary of change
Unrelated questions in forms	Adding conditional logic	Mental health assessment	An example of conditional logic added in the mental health assessment form includes a question asking about the age range of the patient being assessed. Depending on the age range selected, only the form with questions relevant to that population group are displayed to the clinician.
Time taken to complete forms; Duplication within assessment forms	Removing duplication within EHR assessment forms	Risk assessment; physical health assessment	Questions that were duplicated across assessment forms were removed to minimise duplication and reduce time taken to complete forms
Time taken to complete forms	Auto-population of forms	Mental health assessment; Physical health assessment; Risk assessment; HONOS	This feature helped to automatically generate patient information on assessment forms based on previous forms for the same patient within the same care episode. This would allow clinicians to easily see information gathered from previous assessments and edit sections of the form only when an update was necessary. This feature was designed to minimise distress for patients and save time for clinicians.
Time taken to complete forms	Auto-population of letters	Letters to primary care clinicians	This feature helped to automatically generate letters to primary care clinicians from completed assessment forms. Before this change, clinicians would usually type up a separate letter and this would involve duplicating information that had already been added into the assessment forms. Bespoke templates were created to tailor letters from each clinical team with features such as headers and logos. This feature was added to save time for clinicians and increase the likelihood that EHR assessment forms will be completed.
Difficulty navigating the EHR system; Time taken to complete forms	Visual workflow dashboard	Mental health assessment; Physical health assessment; Risk assessment; Patient care plan; Letter to the primary care clinician	A visual workflow dashboard of assessment forms was added. The dashboard consisted of hyperlinks for all assessment forms that would need to be completed for a patient. Prior to this, clinicians would have to manually search for all the forms that needed to be completed. Each row on the assessment dashboard referred to a new patient from the clinician’s caseload. The dashboard was also colour coded using red, amber, green and grey. The dashboard was designed to reduce time and effort when completing assessment forms and improve clinician workflow.

**Measures**

The updated EHR assessment forms were evaluated by comparing the following three measures at baseline and post-changes: (1) usability testing to monitor time and duplication, (2) clinician experience survey, and (3) proportion of completed EHR assessment forms.

**Usability testing**

Usability testing was conducted with three clinicians from one team. The same clinicians were tested at baseline and post-changes to EHR assessment forms. Three hypothetical patient profiles were developed by a consultant psychiatrist (DM) and were similar in the amount of information they contained, the number of words and the level of complexity. Each clinician was randomised to one patient profile at baseline and to either of the remaining profiles post-changes. Clinicians were required to complete all assessment forms for a patient on the EHR system and generate a letter to the primary care clinician, and the following information was recorded: time taken to complete forms, the number of duplication, and the time taken to duplicate information. Clinicians also completed the validated System Usability Scale (SUS) post-testing, which assess satisfaction with usability and consists of 10 items each scored on a 5-point Likert scale (1 = strongly disagree, 5 = strongly agree) [31].

**Clinician experience survey**

An author-developed survey, adapted from previous studies, assessed clinician’s experience of using the EHR assessment forms. The survey questions tapped onto three main components: ease of use, perceived usefulness and user satisfaction [7, 26, 32, 33]. Additional questions assessed clinician’s reported usage and satisfaction with each EHR assessment form. Different members of both clinical teams completed the survey at baseline and after EHR changes were made, as clinicians were conveniently sampled based on who was available in the team when the surveys were distributed.

**Proportion of completed EHR assessment forms**

The number of completed EHR assessment forms by both teams at baseline and post-changes were collected using routinely available data. This was divided by the number of new patient episodes that were open at baseline and post-changes to calculate the proportion of completed EHR assessment forms.

**Statistical analysis**

All statistical analysis was conducted using SPSS. For usability testing, time taken to complete EHR assessment forms, number of duplications and time spent duplicating patient information were compared for differences at baseline and post-changes using descriptive statistics. For the clinician experience, demographics of participants at baseline and post-changes was compared for differences using a chi-squared test and the total composite survey score was compared for differences using an independent t-test. Proportion of completed NHS assessment forms at baseline and post-changes were compared using the two proportion z-tests. Bonferroni correction was applied due to multiple comparisons.

## Results

### Usability testing

#### Demographics

Participant demographics for the three clinicians participating in usability testing were as follows: gender (female, n = 2; male, n = 1), age (30–40 years, n = 2; 50–60 years, n = 1), and profession (doctor, n = 1; psychiatric nurse, n = 1; social worker, n = 1).

#### Time taken to complete assessment forms

At baseline, the average time taken to complete all EHR assessment forms was 62.06 min (SD 14.37 min, range 51.29–82.38 min) and after changes to EHR assessment forms was 36.67 min (SD 13.34 min, range 27.23–46.10 min). There was a decrease of 40.9% in time taken to complete EHR assessment forms.

#### Duplication of assessment forms

Clinicians reported an average of 19 duplications at baseline (SD 4.93; range 3–12) and an average of 3 duplication post-changes (SD = 3; range 0–3). At baseline, the average time spent duplicating patient information was 14.36 min (SD = 10.28 min, range 2.14-13.34 min) and at post-changes was 4.10 min (SD = 0.13 min, range 4-4.19). There was an overall decrease of 71.4% in the time spent duplicating patient information with the updated EHR assessment forms.

#### Post-usability testing

The SUS was administered immediately after usability testing. The average total SUS score at baseline was 19 (SD = 2.64, range 16–21) and at post-changes was 42 (SD = 1.42; range 41–43). There was increased satisfaction with the usability of the updated EHR assessment forms.

### Clinician Experience Survey

#### Demographics

A total of 71 participants completed the survey from both teams (pre-changes, n = 43; post-changes, n = 28). Key demographic information is reported in Table 3. This table shows that there were no significant differences between the group of clinicians completing the survey at baseline and post-changes.

**Table 3:** Demographic information for clinicians completing the clinician experience survey, pre and post changes

	Pre changes (n = 43)	Post changes (n = 28)	Group differences (Chi -square; p-value)
**Feasibility study teams**			**χ** ^**2**^ **(1) = 0.04; p = 0.95**
Team A	**12 (28%)**	**8 (29%)**	
Team B	**31 (72%)**	**20 (71%)**	
**Age**			**χ** ^**2**^ **(4) = 4.18; p = 0.38**
18–30 years old	**6 (14%)**	**8 (29%)**	
31–40 years old	**13 (30%)**	**9 (32%)**	
41–50 years old	**14 (33%)**	**7 (25%)**	
51–60 years old	**7 (16%)**	**4 (14%)**	
> 60 years old	**3 (7%)**	**0**	
**Gender**			**χ** ^**2**^ **(2) = 1.06; p = 0.59**
Female	**26 (60%)**	**20 (72%)**	
Male	**14 (33%)**	**6 (21%)**	
Missing	**3 (7%)**	**2 (7%)**	
**Profession**			**χ** ^**2**^ **(7) = 4.71; p = 0.70**
Administrator	**2 (5%)**	**0**	
Care Coordinator	**25 (58%)**	**17 (60%)**	
Student Nurse	**1 (2%)**	**1 (4%)**	
Consultant Psychiatrist	**2 (5%)**	**0**	
Psychiatrist	**7 (16%)**	**8 (28%)**	
Personal assistant	**1 (2%)**	**0**	
Support and Recovery Worker	**2 (5%)**	**0**	
Therapist	**1 (2%)**	**1 (4%)**	
Missing	**2 (5%)**	**1 (4%)**	
**Years of EHR experience**			**χ** ^**2**^ **(4) = 1.51; p = 0.68**
< 1 year	**8 (18%)**	**4 (14%)**	
1–2 years	**7 (16%)**	**4 (14%)**	
2–5 years	**11 (25%)**	**11 (40%)**	
> 5 years	**17 (39%)**	**9 (32%)**	
Missing	**1 (2%)**	**0**	

EHR = Electronic Health Record; All data reported as n(%) unless stated; Pre-changes =  two months before new EHR assessment forms were implemented; post-changes = within ten weeks after new EHR assessment forms were implemented

### Clinician’s experience of EHR assessment forms

A composite survey score was calculated by summing clinician’s score on each survey item (see Table 4). The average composite survey at baseline was 32.2 (SD 6.6) and post-changes was 41.1 (SD 7.8). Since the data met the assumption of normality (Shapiro-Wilk test at p > 0.005) and equal variance (Levine’s test, F = 2.78, p = 0.1), an independent t-test was conducted and showed a significant difference between pre and post groups, t (67) = -3.005, p = 0.004, 95% CI -8.66 to -1.74. Clinicians had a significantly better experience of completing EHR assessment forms following usability improvements.

#### Clinician’s self-reported usage and satisfaction of EHR assessment forms

Clinician’s reported the highest increase in usage and satisfaction for mental health assessment forms after usability improvements were made (see appendix Table A.1).

**Table 4:** Clinician’s scores on each item of the clinician experience survey and composite survey score, pre and post changes

Item no.	Item description	Pre-changes (n = 43)		Post-intervention (n = 28)	
		N	M (SD)	N	M (SD)
***Perceived ease of use***					
**1**	EHR^a^ assessment forms are easy to use	42	3.1 (1.0)	27	4.1 (0.5)
**3**	Finding the right assessment forms on EHR system requires a lot of time *	42	3.3 (1.0)	27	3.7 (1.5)
**5**	Difficult to see if EHR assessment forms have been completed *	42	3.3 (1.2)	27	3.7 (1.4)
**6**	Easy to see what EHR assessment form needs to be completed	42	2.2 (1.2)	27	3.9 (1.0)
**8**	EHR assessment forms in EHR system are not worth the time and effort required to use them *	42	2.8 (1.0)	27	4.2 (1.1)
***Perceived usefulness***					
**4**	The assessment forms on EHR system captures the essential patient data for my service	42	3.3 (0.9)	27	4.0 (1.0)
**9**	Prefer to write letter to primary care clinician in Word than use EHR assessment forms *	42	2.8 (1.2)	27	3.9 (1.4)
**10**	The EHR assessment forms limit ability to record important patient information *	42	2.8 (0.9)	27	4.1 (1.2)
***User satisfaction***					
**2**	The quality of EHR assessment forms are good	42	2.9 (0.9)	27	3.7 (0.7)
**7**	I am generally satisfied with the EHR system	42	2.8 (1.0)	27	3.8 (1.2)
**11**	EHR assessment forms are user-friendly	42	2.8 (0.9)	27	3.7 (1.0)
	**Total composite survey score**	42	32.2 (6.6)	27	41.1 (7.8)

EHR = Electronic Health Record; * = scores for negatively worded statements were reversed; ^a^ = EHR was replaced by the name of the EHR system in the current survey; the n for each survey item is reported after missing data; Pre-changes = two months before new EHR assessment forms were implemented; post-changes = within ten weeks after new EHR assessment forms were implemented

### Proportion of completed EHR forms

The number of completed EHR assessment forms at baseline and post-changes, and the results of the two proportion z-tests, are shown in Table 5. Clinician’s use of the following forms increased significantly after usability improvements were made: mental health assessment, risk assessment, HONOS and patient care plans. The physical health assessment forms did not show a significant increase in usage after Bonferroni correction was applied. There was an overall increase in clinician’s use of EHR assessment forms after usability improvements, but the increase in usage of physical health assessment forms was not significant.

**Table 5:** Number of completed EHR assessment forms for new patient episodes opened in both teams and two proportion z-test of differences, pre and post changes

EHR assessment forms	Patient episodes, pre-changes (n = 905)	Patient episodes, post-changes (n = 1116)	Differences (z-test, p-value, 95% CI)
Mental health assessment	147 (16.24%)	307 (27.5%)	X^2^(1) = 35.771, p < 0.001, -0.15 to -0.08
Physical health assessment	39 (4.3%)	72 (6.4%)	X^2^(1) = 8.304, p = 0.04, -0.11 to -0.02
Risk assessment	347 (38.3%)	500 (44.8%)	X^2^(1) = 4.015, p = 0.045, -0.04 to -0.001
HONOS	205 (22.6%)	320 (28.7%)	X^2^(1) = 9.114, p = 0.003, -0.10 to -0.02
Patient care plans	74 (8.2%)	139 (12.5%)	X^2^(1) = 9.254, p = 0.002, -0.07 to -0.02

EHR = Electronic Health Record; HONOS = Health of the Nation Outcome Scale; Pre-changes = two months before new EHR assessment forms were implemented; post-changes = within ten weeks after new EHR assessment forms were implemented

## Discussion

The current study evaluated usability improvements made to existing EHR assessment forms within a mental health setting in the UK. This was a feasibility study conducted with two mental health teams before usability changes were implemented across the wider healthcare organisation. All changes to the EHR system were made in collaboration with clinicians. Evaluation of the updated EHR assessment forms showed that clinicians spent less time completing forms and duplicating patient information, were satisfied with the usability changes, and completed more EHR assessment forms for patients after usability was improved.

**Fig. 2:**
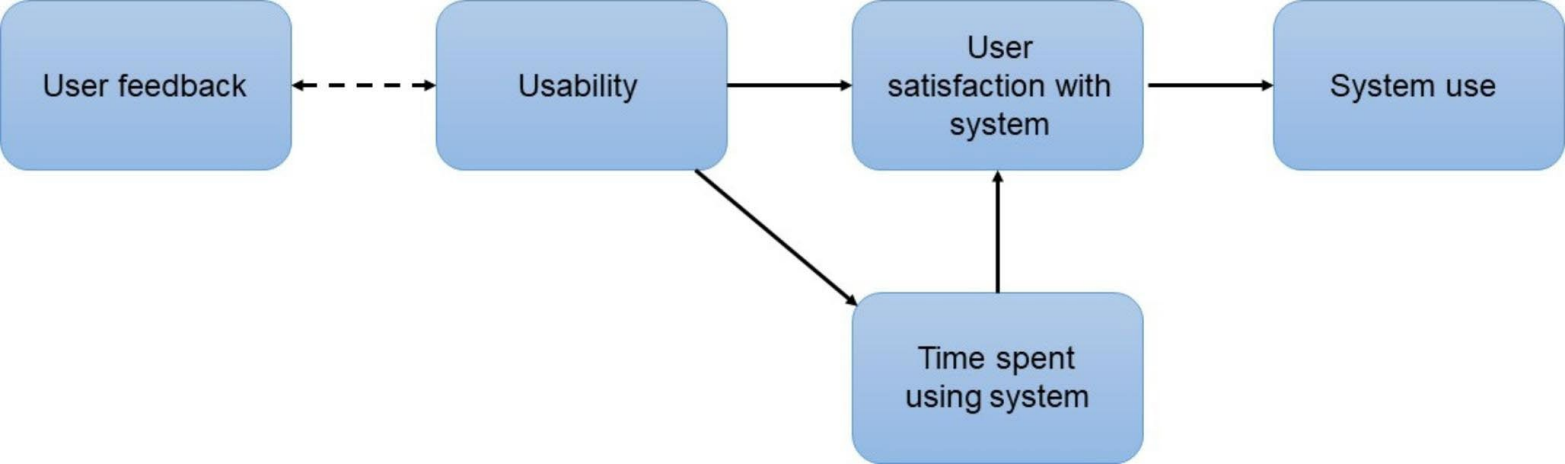
Framework to show relationship between usability and usage of an existing technological system. According to the framework, user feedback can influence the usability of a system. System usability is likely to impact system use, mediated by user satisfaction and time spent using the system

The technology acceptance model proposes that usability of a system can predict adoption of that system and is a widely used model when implementing EHRs into healthcare services [8, 9, 34]. The current findings offer some further insights about how usability can sustain the usage of existing systems. We have developed a framework to conceptualise the results of present and past research findings; we propose that usability of a system can affect the use of existing systems such as EHRs and this is mediated by improved user satisfaction and reduction in unnecessary time spent using the system. This framework also supports the need to engage users when making usability improvements for a better outcome (see Fig. 2).

According to this framework, improving usability would need to improve user satisfaction with a system before usage of the system is increased. In the current study, both user satisfaction and the use of EHR assessment forms increased after usability was improved, but the direction of this relationship is unclear as user satisfaction and use of EHRs were assessed at the same time. However, previous work supports the notion that increased user satisfaction with the system can subsequently lead to increased usage of the system [12, 22]. As proposed in our framework, clinician’s satisfaction with the system could also be mediated by the time spent using the system. Clinicians in the present study were much quicker in completing EHR assessment forms and spent less time duplicating patient information which likely contributed to improved satisfaction. Reducing clinician’s time in using EHRs also has potential long-term implications; information recorded in EHRs is likely to be accurate and complete, clinician’s cognitive workload could be reduced and more clinician time could be spent in direct patient care [12–14]. Longitudinal studies or longer follow-ups are needed to evaluate these possible long-term implications. In terms of usage, there was an overall increase in completed EHR assessment forms in the present study, with only the physical health assessment forms not showing a significant increase. This is a promising finding for a short-term feasibility study. This is also consistent with previous studies which find that when EHRs were difficult to use, clinicians would only partially use EHRs, find their own workarounds in the system or rely only on paper-based forms [14, 15, 22]. However, these practices can compromise the safety and quality of patient care. Thus, improving EHR usability and clinician satisfaction is important to ensure a consistent and accurate record of patient information.

Specific usability changes in the present study could have further impacted clinician’s satisfaction and use of the system. One reason for this could be as these changes may have improved the general clinical workflow, by making EHRs become a meaningful and integral tool to support patient care, rather than a burdensome task for clinicians [35, 36]. Usability changes such as introducing a visual dashboard, removing duplicate questions across forms, and auto-population features were all aimed at making EHR assessment forms quicker and efficient to use, but could have indirectly improved the workflow of the system [12, 17, 22]. For instance, the auto-population feature used in the present study could have specific implications for mental health services. Auto-populating EHRs based on previous information for the same patient could prevent mental health patients from repeatedly recalling psychologically distressing information [16, 18]. Further, auto-population of letters for primary care clinicians could also be used for clinicians from other services to facilitate integration of services for mental health patients [16, 18]. However, the impact of features such as auto-population features on clinical workflow were not directly assessed in the present study and could be evaluated in the future.

As proposed in our framework, user feedback can be helpful when deciding which usability changes should be made to an existing system. In the present study, usability changes were directly tailored to clinicians in the healthcare organisation. The changes also underwent many iterations based on clinician input. This strategy has been widely recommended in previous studies [13, 17, 23, 37]. Encouraging a dialogue between clinicians and the EHR team can also ensure that usability changes are within the limits of technology and help both parties come to a shared understanding about the purpose of the EHR system. Future usability studies should adopt this approach to make the process of change efficient for both clinicians and EHR teams and increase the likelihood that the usability changes will lead to improved outcomes.

### Limitations and future work

The current findings should be interpreted in light of its limitations. First, this was a feasibility study conducted with two mental health teams within a short time-frame and is not easily generalisable. Another limitation was the small sample size of clinicians in the usability testing and the fact that these clinicians were only recruited from one clinical team. While small sample sizes are generally sufficient in highlighting any obvious usability errors, future work should consider including a larger number of clinicians from different clinical teams. Another limitation was the author-developed clinician experience survey which had not been previously validated. To minimise this limitation, questions in the present survey were adapted from previous work and the survey was used in combination with other evaluation methods. The framework examining how usability could have an impact on usage of existing systems could not be verified within the current study. Controlled experimental studies or longitudinal studies using an interrupted time series methodology are needed to further validate this framework [38, 39]. Finally, the content of the EHR assessment forms themselves can also impact clinician’s satisfaction and usage of the forms. However, this was beyond the scope of the current work but should be considered in the future.

## Conclusions

Poor EHR usability can be a barrier for clinicians to use EHRs consistently and accurately. There is limited research on how to improve EHR usability in mental health services. The current feasibility study made usability changes to an existing EHR system by reducing duplication, improving navigation, customising forms to clinical teams and adding auto-population features. The results showed that improving usability of EHRs reduced clinician’s time spent completing and duplicating patient information, improved clinician’s satisfaction with the system and increased usage of EHR assessment forms in the clinical service. It is important to tailor EHR usability improvements to clinicians who are users of the system. Future work should improve EHR usability across the wider healthcare organisation and evaluate the longer-term implications for clinician’s workload and patient care in mental health settings.

## Electronic supplementary material

Below is the link to the electronic supplementary material.

Supplementary Material 1

Supplementary Material 2

Supplementary Material 3

## Appendix

**Figure A.1.** Example of a new EHR mental health assessment form for adult patients, with conditional logic.

‘Service setting’ question used as a conditional logic in the form.

**Figure A.2** Example of a new EHR mental health assessment form for children and adolescent patients, with conditional logic.

‘Service setting’ question used as a conditional logic in the form.

**Figure A.3.** Visual workflow dashboard for new EHR assessment forms.

The visual dashboard for clinician caseload with patient information removed; Dates refer to when the form was last completed or saved.

**Table A.1:** Clinician’s reported usage and satisfaction with specific EHR assessment forms, pre and post changes

EHR assessment forms	Pre-changes (n = 43)		Post-changes (n = 28)	
	n	M (SD)	n	M (SD)
**Frequency of use**				
Mental Health Form	43	2.3 (1.6)	27	3.7 (1.1)
Risk Assessment Form	43	4.3 (1.0)	28	4.4 (1.2)
Physical Health Form	41	2.3 (1.1)	27	2.6 (1.2)
Patient care plan	43	2.9 (1.6)	27	2.6 (1.4)
Letter to the primary care clinician	N/A	N/A (N/A)	27	2.5 (1.5)
**Satisfaction**				
Mental Health Form	41	3.0 (1.0)	27	3.8 (1.3)
Risk Assessment Form	42	3.6 (1.0)	27	4.0 (1.0)
Physical Health Form	39	3.1 (1.1)	27	2.8 (0.9)
Patient care plan	40	3.6 (0.8)	28	3.3 (0.9)
Letter to the primary care	N/A	N/A	23	3.2 (1.0)
Visual dashboard	N/A	N/A	26	3.7 (1.1)

EHR = Electronic Health Record; the n for each survey item is reported after missing data; Pre-changes = two months before new EHR assessment forms were implemented; Post-changes = ten weeks after new EHR assessment forms were implemented
