# Correction to: Improving usability of Electronic Health Records in a UK Mental Health Setting: A Feasibility Study

**DOI:** 10.1007/s10916-022-01848-6

**Published:** 2022-08-13

**Authors:** Ruta Buivydaite, Gurpreet Reen, Tatjana Kovalevica, Harry Dodd, Ian Hicks, Charles Vincent, Daniel Maughan

**Affiliations:** 1 Department of Experimental Psychology, University of Oxford, Oxford, UK; 2 Oxford Health NHS Foundation Trust, Oxford, UK

## Correction to: Journal of Medical Systems (2022) 46:50 https://doi.org/10.1007/s10916-022-01832-0

The equal contribution statement for authors Ruta Buivydaite and Gurpreet Reen is missing in the original publication of this article. The original article has been corrected.

