# Supplementary material for: Improving usability of Electronic Health Records in a UK Mental Health setting: a feasibility study
Source: J Med Syst. 2022 Jun 8;46(7):50. doi: 10.1007/s10916-022-01832-0 (PMC9177469; doi:10.1007/s10916-022-01832-0)
Supplement: Supplementary file 1 — Supplementary Material 1 [file 10916_2022_1832_MOESM1_ESM.pdf]

Hide Menu Carenotes

User: Daniel

TEST, XX TEST

Born 20/05/198[illegible]

Gende[illegible]

Addr. No Fixed Abode, ZZ99 3VZ

NHS No. Unl[illegible]

GP	Dr. Not Applicable	Patient ID	371021		
Consultant		Gender	Male	Care Type	No CPA
Primary Worker		Legal Status	No MHA Section		
				Assessment MH	
Alert	No Alerts recorded against this patient				

Status			
Unconfirmed			
Date		Time	
Confirmed By		Job Title	

Form Details			
Original Author*	Daniel Maughan		
Entered Date	07/04/2020	Entered Time	12:25
Event Date*	07/04/2020	Event Time*	12:25

The User Guide for the completion of this form is available on the Trust Intranet here

Service Setting	
See Additions information for each service, CYP: Adult: Older Adult: Learning Disabilities: Perinatal	
**Service***	○ CYP ◉ Adult ○ Older People ○ Learning Disabilities ○ Perinatal

Generic Assessment	Please check the relevant assessments before starting this form.
**Presenting Situation including the Patients view of Difficulties**	**Adult/Perinatal**
**History of Presenting Complaint**	**Adult/Perinatal**
**Medical History**	**Adult/Perinatal**
**Current Medication**	**Adult/Perinatal**
**Psychiatric History**	**Adult/Perinatal**
**Personal History**	**Adult/Perinatal**
**Family History**	**Adult/Perinatal**
**Forensic History**	**Adult/Perinatal**

Social Circumstances	Adult/Perinatal
Alcohol and Drug Usage	Adult/Perinatal
Spiritual/Cultural Needs	Adult/Perinatal
Is the Patient a Carer, Parent or being cared for?	Adult/Perinatal
Pre-Morbid Personality	Adult/Perinatal
Safeguarding Status*	Adult/Perinatal Adult policy<br>○ There is Safeguarding Involvement ○ Safeguarding concerns ○ No safeguarding concerns or issues
Does the Patient have any dependents?*	Adult/Perinatal<br>○ Yes ○ No ◉ Not Assessed
Capacity at time of Assessment	Adult/Perinatal

Mental State Examination	
Appearance	
Behaviour	
Speech	
Mood	
Thoughts	
Perception	
Cognition	
Insight	

For further guidance on the statutory duties of the Care Act here

Care Act Compliance	To ascertain if this person has a significant long term unmet social care need affecting their wellbeing, which will addressed by their mental health treatment please complete the questions below.
Is the person having problems with managing and maintaining nutrition?*	○ Yes ○ No ○ Not Known ◉ Not Assessed
	○ Yes ○ No ○ Not Known ◉ Not Assessed

Is the person having problems maintaining personal hygiene?*	
Is the person having problems managing toilet needs?*	○ Yes ○ No ○ Not Known ◉ Not Assessed
Is the person having problems being appropriately clothed?*	○ Yes ○ No ○ Not Known ◉ Not Assessed
Is the person having problems being able to make use of the adults home safely?*	○ Yes ○ No ○ Not Known ◉ Not Assessed
Is the person having problems maintaining a habitable home environment?*	○ Yes ○ No ○ Not Known ◉ Not Assessed
Is the person having problems developing and maintaining family or other personal relationships?*	○ Yes ○ No ○ Not Known ◉ Not Assessed
Is the person having problems accessing and engaging in work, training, education or volunteering?*	○ Yes ○ No ○ Not Known ◉ Not Assessed
Is the person having problems making use of necessary facilities or services in the local community, including public transport, and recreational facilities or services?*	○ Yes ○ No ○ Not Known ◉ Not Assessed
Is the person having problems carrying out any caring responsibilities the adult has for a child?*	○ Yes ○ No ○ Not Known ◉ Not Assessed

Psychological Therapies	
Is a referral to Psychological Therapies needed?*	○ Yes ○ No ○ Not asked ◉ Not Assessed

Third Party Information	
Consider carer, parent, relative, any other involved in patient's care. Include any specific requests to who should/should not receive information.	

Actions	
Medication Changes*	○ Yes ○ No ○ Not Known ◉ Not Applicable
Actions for GP*	○ Yes ○ No ○ Not Known ◉ Not Applicable
Follow up clinical review required?*	○ Yes ○ No ○ Not Known ◉ Not Applicable **Ensure you record the next clinical using the diary appoi form**
Additional Plans*	**Adult/Perinatal** ⓘ<br>○ Yes ○ No ○ Not Known ◉ Not Applicable

Research and Consent	
Has the Patient consented to be contacted by Research?*	○ Yes ○ No ○ Not Asked
Does the Patient want a copy of the Assessment letter?*	○ Yes ○ No ○ Not Asked
Is there anyone else that should receive a copy?*	○ Yes ○ No ○ Not Asked

Information Governance message to all users of patient information
The Data Protection Act and GDPR means that patients have important and extensive rights around what the Trust does with their data. By accessing you are processing data on behalf of the Trust and you have personal legal responsibilities Click here. Use of patient records must comply with Trust Information Governance Policy (Click here), Standard Operating Procedures (Click here, access the IG Tab) and Data Protection Act 2018 (Clic
Respect information and privacy.
