# Supplementary material for: Improving usability of Electronic Health Records in a UK Mental Health setting: a feasibility study
Source: J Med Syst. 2022 Jun 8;46(7):50. doi: 10.1007/s10916-022-01832-0 (PMC9177469; doi:10.1007/s10916-022-01832-0)
Supplement: Supplementary file 2 — Supplementary Material 2 [file 10916_2022_1832_MOESM2_ESM.pdf]

Hide Menu Carenotes User: Daniel

TEST, XX (Mr) *Born* **14/11/2018 (16**

Gende

Addr. **No Fixed Abode, ZZ99 3VZ** *NHS No.* **Unl**

GP	Dr Unknown	Patient ID	352982		
Consultant		Gender	Male	Care Type	No CPA
Primary Worker		Legal Status	No MHA Section	Assessment MH	
		School	No school recorded		
Alert	No Alerts recorded against this patient				

Status			
Unconfirmed			
Date		Time	
Confirmed By		Job Title	

Form Details			
Original Author*	Daniel Maughan		
Entered Date	07/04/2020	Entered Time	12:23
Event Date*	07/04/2020	Event Time*	12:23

The User Guide for the completion of this form is available on the Trust Intranet here

Service Setting	
See Additions information for each service, CYP: Adult: Older Adult: Learning Disabilities: Perinatal	
**Service***	(●) CYP ( ) Adult ( ) Older People ( ) Learning Disabilities ( ) Perinatal

Generic Assessment	Please check the relevant assessments before starting this form.
**Presenting Situation including the Patients view of Difficulties**	**CYP**
**History of Presenting Complaint**	**CYP**
**Medical History**	**CYP**
**Current Medication**	**CYP**
**Psychiatric History**	**CYP**
**Personal History**	**CYP**
**Family History**	**CYP**
**Forensic History**	**CYP**

Social Circumstances	CYP
Alcohol and Drug Usage	CYP
Spiritual/Cultural Needs	CYP
Is the Patient a Carer, Parent or being cared for?	CYP
Pre-Morbid Personality	CYP
Safeguarding Status*	CYP Children's policy<br>○ There is Safeguarding Involvement ○ Safeguarding concerns ○ No safeguarding concerns or issues
Does the Patient have any dependents?*	CYP<br>○ Yes ○ No ◉ Not Assessed
Capacity at time of Assessment	CYP

Mental State Examination	
Appearance	
Behaviour	
Speech	
Mood	
Thoughts	
Perception	
Cognition	
Insight	

Additional Information	
Eating behaviours	

	ℹ
**School**	
	ℹ
**Engagement**	
	ℹ
**Developmental History**	
	ℹ
**Observation of Behaviour**	

Third Party Information	
**Consider carer, parent, relative, any other involved in patient's care. Include any specific requests to who should/should not receive information.**	

Actions		
**Medication Changes***	○ Yes ○ No ○ Not Known ◉ Not Applicable	
**Actions for GP***	○ Yes ○ No ○ Not Known ◉ Not Applicable	
**Follow up clinical review required?***	○ Yes ○ No ○ Not Known ◉ Not Applicable	**Ensure you record the next clinical using the diary appoi form**
	**CYP** ℹ	
**Additional Plans***	○ Yes ○ No ○ Not Known ◉ Not Applicable	

Research and Consent	
**Has the Patient consented to be contacted by Research?***	○ Yes ○ No ○ Not Asked
**Does the Patient want a copy of the Assessment letter?***	○ Yes ○ No ○ Not Asked
**Is there anyone else that should receive a copy?***	○ Yes ○ No ○ Not Asked

Information Governance message to all users of patient information
The Data Protection Act and GDPR means that patients have important and extensive rights around what the Trust does with their data. By accessinç you are processing data on behalf of the Trust and you have personal legal responsibilities Click here. Use of patient records must comply with Trus Information Governance Policy (Click here), Standard Operating Procedures (Click here, access the IG Tab) and Data Protection Act 2018 (Clic
Respect information and privacy.
