# Supplementary figures and images for: Improving usability of Electronic Health Records in a UK Mental Health setting: a feasibility study
Source: J Med Syst. 2022 Jun 8;46(7):50. doi: 10.1007/s10916-022-01832-0 (PMC9177469; doi:10.1007/s10916-022-01832-0)

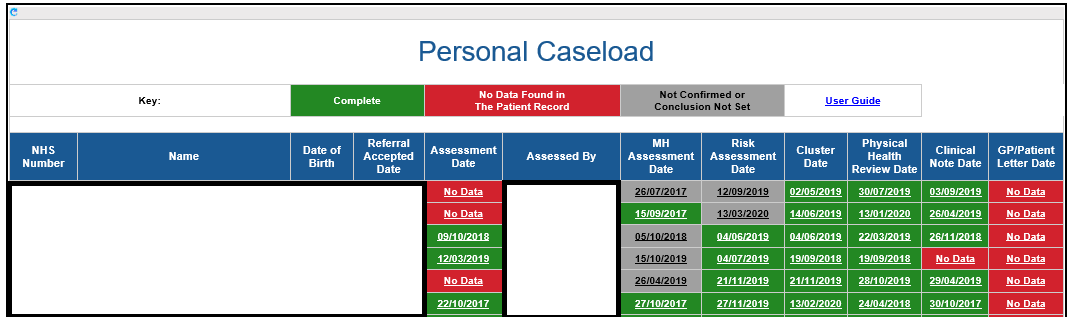

Supplement: Supplementary file 3 — Supplementary Material 3 [file 10916_2022_1832_MOESM3_ESM.png]
